# The nitrilase PtNIT1 catabolizes herbivore-induced nitriles in *Populus trichocarpa*

**DOI:** 10.1186/s12870-018-1478-z

**Published:** 2018-10-22

**Authors:** Jan Günther, Sandra Irmisch, Nathalie D. Lackus, Michael Reichelt, Jonathan Gershenzon, Tobias G. Köllner

**Affiliations:** 1 Max Planck Institute for Chemical Ecology, Hans-Knöll-Strasse 8, D-07745 Jena, Germany; 2 Present Address: Michael Smith Laboratories, University of British Columbia, Vancouver, Canada

**Keywords:** *Populus trichocarpa*, Nitrilase, NIT1, β-Cyanoalanine, Benzyl cyanide, Plant defense

## Abstract

**Background:**

Nitrilases are nitrile-converting enzymes commonly found within the plant kingdom that play diverse roles in nitrile detoxification, nitrogen recycling, and phytohormone biosynthesis. Although nitrilases are present in all higher plants, little is known about their function in trees. Upon herbivory, poplars produce considerable amounts of toxic nitriles such as benzyl cyanide, 2-methylbutyronitrile, and 3-methylbutyronitrile. In addition, as byproduct of the ethylene biosynthetic pathway upregulated in many plant species after herbivory, toxic β-cyanoalanine may accumulate in damaged poplar leaves. In this work, we studied the nitrilase gene family in *Populus trichocarpa* and investigated the potential role of the nitrilase PtNIT1 in the catabolism of herbivore-induced nitriles.

**Results:**

A BLAST analysis revealed three putative nitrilase genes (*PtNIT1*, *PtNIT2*, *PtNIT3*) in the genome of *P. trichocarpa*. While *PtNIT1* was expressed in poplar leaves and showed increased transcript accumulation after leaf herbivory, *PtNIT2* and *PtNIT3* appeared not to be expressed in undamaged or herbivore-damaged leaves. Recombinant PtNIT1 produced in *Escherichia coli* accepted biogenic nitriles such as β-cyanoalanine, benzyl cyanide, and indole-3-acetonitrile as substrates in vitro and converted them into the corresponding acids. In addition to this nitrilase activity, PtNIT1 showed nitrile hydratase activity towards β-cyanoalanine, resulting in the formation of the amino acid asparagine. The kinetic parameters of PtNIT1 suggest that the enzyme utilizes β-cyanoalanine and benzyl cyanide as substrates in vivo. Indeed, β-cyanoalanine and benzyl cyanide were found to accumulate in herbivore-damaged poplar leaves. The upregulation of ethylene biosynthesis genes after leaf herbivory indicates that herbivore-induced β-cyanoalanine accumulation is likely caused by ethylene formation.

**Conclusions:**

Our data suggest a role for PtNIT1 in the catabolism of herbivore-induced β-cyanoalanine and benzyl cyanide in poplar leaves.

**Electronic supplementary material:**

The online version of this article (10.1186/s12870-018-1478-z) contains supplementary material, which is available to authorized users.

## Background

Nitrilases are thiol enzymes that have gained attention in plant biology over the last 50 years for their roles in plant development [1], detoxification of nitriles originating from other metabolic processes [2], and nitrogen recycling [3]. Plant nitrilases are members of the nitrilase superfamily that is classified into 13 branches of enzymes [4]. Most of these branches consist of enzymes that catalyze the hydrolysis of nitrile or amide bonds. The nitrilase branch (branch 1, EC 3.5.5.1) comprises enzymes that are capable of accepting nitriles as substrate. The assignment of enzymes to the nitrilase branch relies strongly on the identity of the amino acids flanking the catalytic triad Glu-Lys-Cys [4]. Usually, plants possess multiple copies of nitrilase genes (reviewed in [3]). The thale cress *Arabidopsis thaliana,* for example, contains 4 functional nitrilases [5, 6] that can be divided into 2 different groups, namely the β-cyanoalanine nitrilases (NIT4 group) [7] and indole-3-acetonitrile nitrilases (NIT1 group) [6]. Homologues of these groups have been identified in several species of higher plants [3].

Members of the NIT1 group are known to convert indole-3-acetonitrile (IAN) to the auxin indole-3-acetic acid (IAA) in vitro [8], even though the biological relevance of the biosynthesis of IAA from IAN via a nitrilase pathway in planta is still a subject of discussion [1, 9, 10]. In the Brassicaceae and Poaceae, however, NIT1 homologues were also shown to be involved in the metabolism of breakdown products of glucosinolates [6] and cyanogenic glycosides [11], respectively. Members of the NIT4 group are of widespread occurrence in the plant kingdom. They were shown to convert β-cyanoalanine to asparagine and aspartic acid [12] and are thus termed nitrilase/α-cyano hydrolases [3]. The NIT4 substrate β-cyanoalanine is commonly produced as a detoxification product of hydrogen cyanide, which is either synthesized as byproduct of ethylene biosynthesis [13] or formed as a breakdown product of cyanogenic compounds such as cyanogenic glycosides and cyanogenic lipids [14, 15].

While nitrilases have been investigated in herbaceous plants and grasses, little is known about their occurrence and function in woody plants. Irmisch and colleagues [16] showed that the model tree species *Populus trichocarpa* biosynthesizes considerable amounts of volatile nitriles upon herbivory, such as benzyl cyanide, 2-methylbutyronitrile, and 3-methylbutyronitrile. These nitriles are produced from their respective amino acid precursors through the action of cytochrome P450 monooxygenases from the CYP79 and CYP71 families. Poplar CYP79D6 and CYP79D7 were reported to convert amino acids into aldoximes [16], which are then accepted as substrates by CYP71B40/41 to produce the corresponding nitriles [17]. Olfactometer experiments revealed a strong repellent effect of these volatile nitriles against gypsy moth caterpillars, which indicates a role for these compounds in direct plant defense [17]. RNAi-mediated down regulation of *CYP79D6/7* in *P. x canescens* and the overexpression of *CYP79D6* in *Nicotiana benthamiana* additionally suggested that herbivore-induced nitriles can be further metabolized into aldehydes and alcohols in poplar and other plant species [16].

Since many plants are known to produce ethylene in response to herbivory [18], it is reasonable to speculate that herbivore feeding induces the production of hydrogen cyanide and subsequently β-cyanoalanine in poplar. The formation of the plant hormone ethylene and its byproduct hydrogen cyanide are often associated with abiotic and biotic stresses (reviewed in [19]) and it has been reported that the toxic hydrogen cyanide can even convey a fitness benefit to ethylene-producing plants by increasing pathogen resistance [20]. However, the formation and accumulation of β-cyanoalanine and toxic benzyl cyanide imply the need of nitrilase enzymes that can detoxify the herbivore-induced nitriles to prevent autotoxicity to the plant. In this study, we show that benzyl cyanide can be converted into phenylacetic acid in herbivore-damaged poplar leaves. Moreover, we report the identification and biochemical characterization of the nitrilase PtNIT1 from *P. trichocarpa*. Heterologous expression and kinetic characterization of the recombinant enzyme as well as transcript accumulation analysis by quantitative real time PCR revealed a role for PtNIT1 in the catabolism of herbivore-induced β-cyanoalanine and benzyl cyanide in poplar.

## Results

### β-Cyanoalanine and benzyl cyanide accumulate upon herbivory in poplar leaves

**Fig. 1:**
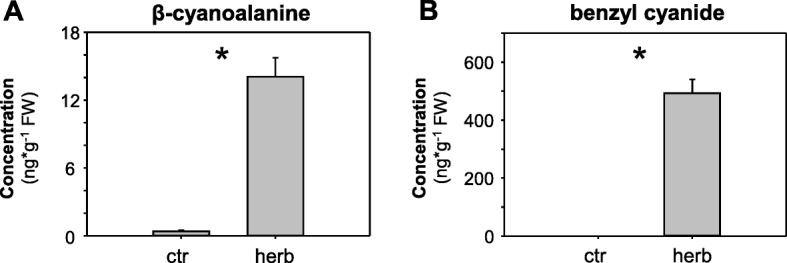
β-Cyanoalanine (**a**) and benzyl cyanide (**b**) accumulate after herbivore feeding on *Populus trichocarpa* leaves. *L. dispar-*damaged leaves (herb) and undamaged control leaves (ctr) were extracted with methanol (**a**) or hexane (**b**) and analyzed via LC-MS/MS and GC-MS, respectively. Means and SE are shown (*n* = 10 biological replicates). Significant differences between the two treatments in Student’s t-tests are indicated (asterisks; *p* < 0.001). FW, fresh weight. Other nitriles such as 2-methylbutyronitrile and 3-methylbutyronitrile that have been described as components of the herbivore-induced volatile blend of *P. trichocarpa* [16] could not be detected in the hexane extracts

Recently it has been shown that caterpillar feeding induces the formation and emission of volatile nitriles such as benzyl cyanide, 2-methylbutyronitrile, and 3-methylbutyronitrile in poplars [16, 17]. However, whether volatile nitriles or other nitriles such as β-cyanoalanine accumulate in herbivore-damaged poplar leaves to any significant degree has remained unclear. To determine the concentration of these compounds in poplar, we measured β-cyanoalanine, benzyl cyanide, 2-methylbutyronitrile, and 3-methylbutyronitrile in methanol extracts and hexane extracts, respectively, made from herbivore-damaged and undamaged *P. trichocarpa* leaves. Both, β-cyanoalanine and benzyl cyanide were barely detectable in undamaged control leaves, but showed a significantly increased accumulation in leaves after feeding of *Lymantria dispar* caterpillars (Fig. 1). 2-Methylbutyronitrile and 3-methylbutyronitrile, however, could not be detected in the extracts of damaged or undamaged leaves, suggesting that these aliphatic nitriles are more volatile than benzyl cyanide and thus do not accumulate in detectable concentrations in the plant.

### Benzyl cyanide can be converted to phenylacetic acid in planta

**Fig. 2:**
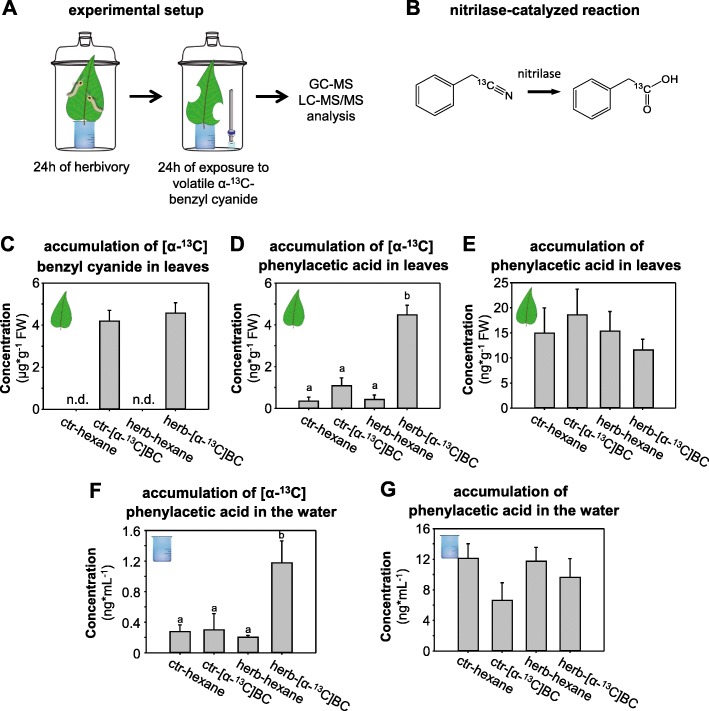
Benzyl cyanide can be converted into phenylacetic acid in vivo in herbivore-damaged *Populus trichocarpa* leaves. Single leaves of *P. trichocarpa* were placed in water and damaged by *L. dispar*-feeding for 24 h, followed by a 24 h exposure either to volatile [α-^13^C]benzyl cyanide dissolved in hexane or to pure hexane (**a**). The conversion of [α-^13^C]benzyl cyanide into [α-^13^C]phenylacetic acid is likely catalyzed by a nitrilase (**b**). Accumulated [α-^13^C]benzyl cyanide was extracted from leaf material with hexane and analyzed using GC-MS (**c**). ^13^C-labeled (**d**) and unlabeled (**e**) phenylacetic acid were extracted from leaf material with methanol and measured using LC-MS/MS. Labeled (**f**) and unlabeled (**g**) phenylacetic acid could also be detected in the water, in which leaf petioles were placed during the experiment. Means and SE are shown (*n* = 5 biological replicates). A two way ANOVA with square root-transformed raw data was performed to test for significance (*p* < 0.05). ctr-hexane, undamaged leaves exposed to hexane; ctr-[α-^13^C]BC, undamaged leaves exposed to volatile [α-^13^C]benzyl cyanide dissolved in hexane; herb-hexane, herbivore-damaged leaves exposed to hexane; herb-[α-^13^C]BC, herbivore-damaged leaves exposed to volatile [α-^13^C]benzyl cyanide dissolved in hexane

While the nitrile β-cyanoalanine has been frequently reported as a nitrilase substrate in various plant species [3, 5, 21], little is known about the metabolic fate of benzyl cyanide and its potential conversion by nitrilases in planta. Piotrowski and colleagues [3, 11] showed that AtNIT4 from *Arabidopsis thaliana* and SbNIT4A from *Sorghum bicolor* accept, among others, benzyl cyanide as substrate in vitro, but whether this compound can be converted to phenylacetic acid in vivo is unclear. In this study we investigated the capability of poplar to metabolize exogenous [α-^13^C]benzyl cyanide. Detached leaves of *P. trichocarpa* were placed in water and subjected to *L. dispar* caterpillar feeding. Subsequently, the leaves were exposed to volatile [α-^13^C]benzyl cyanide and the uptake and accumulation of this potential nitrilase substrate as well as the formation of ^13^C-labeled and unlabeled phenylacetic acid were measured using gas chromatography-mass spectrometry and liquid chromatography-mass spectrometry, respectively (Fig. 2a). Both undamaged and herbivore-damaged leaves that were exposed to volatile [α-^13^C]benzyl cyanide accumulated significant and equivalent amounts of this compound, indicating an efficient uptake from the headspace (Fig. 2c). Conversion of [α-^13^C]benzyl cyanide to [α-^13^C]phenylacetic acid occurred in both treatments. However, the accumulation of the labeled acid was significantly higher in herbivore-damaged leaves in comparison to undamaged controls (Fig. 2d). The small amounts of [α-^13^C]phenylacetic acid that were detected in undamaged and herbivore-damaged leaves not treated with ^13^C-labeled benzyl cyanide are likely due to naturally occurring *m* + 1 isotopes of unlabeled phenylacetic acid. Interestingly, labeled and unlabeled phenylacetic acid could also be detected in the water in which leaf petioles were placed during the experiment, and the observed secretion patterns were highly similar to those detected in leaves (Fig. 2f-g). Altogether these data suggest the presence of herbivore-induced nitrilase activity in poplar with benzyl cyanide as substrate as well as the potential transport of phenylacetic acid from the leaves to other plant organs.

### *Populus trichocarpa* possesses three putative nitrilase genes

**Fig. 3:**
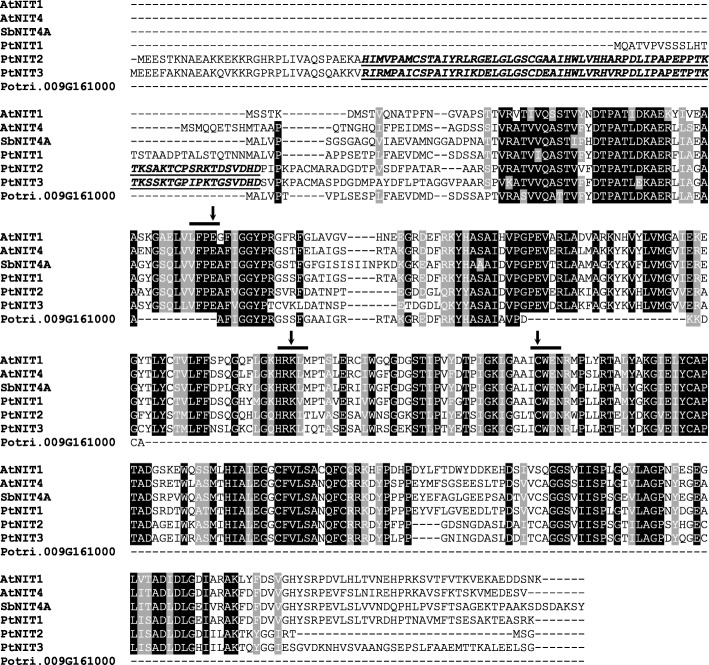
Amino acid alignment of *Populus trichocarpa* nitrilases, PtNIT1, PtNIT2, PtNIT3, and Potri.009G161000 with SbNIT4A from *Sorghum bicolor* and AtNIT1/4 from Arabidopsis. Black boxes mark conserved residues and grey boxes mark residues with similar physicochemical properties. Residues forming the catalytic triad are highlighted with arrows. Sequence motifs that are conserved throughout the nitrilase family are marked by black lines. The TCP domains of PtNIT2 and PtNIT3 are underlined and written in italics

**Fig. 4:**
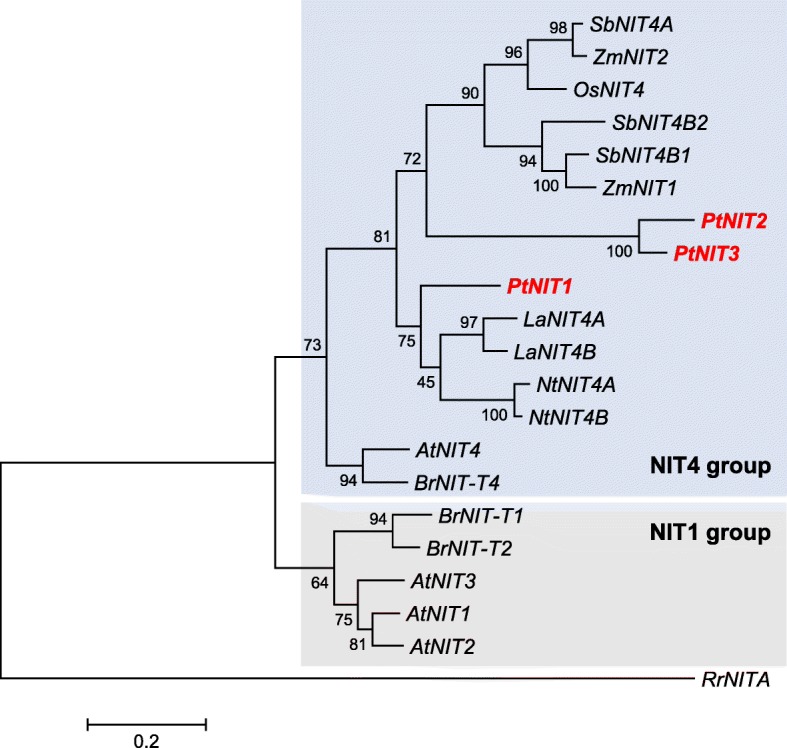
Dendrogram analysis of poplar nitrilase genes and characterized nitrilase genes from other plants. The tree was inferred by using the maximum likelihood method and *n* = 1000 replicates for bootstrapping. Bootstrap values are shown next to each node. *Rhodococcus rhodochrous* nitrilase was used as outgroup. The tree is drawn to scale, with branch lengths measured as the number of substitutions per site. Genes described in this study are shown in red and bold. Accession numbers: *AtNIT4* (AT5G22300); *BrNIT-T4* (ABM55735); *PtNIT1* (Potri.004G199600); *PtNIT2* (Potri.006G207700); *PtNIT3* (Potri.016G074200); *SbNIT4B2* (Sb04g026940); *SbNIT4B1* (Sb04g026930); *ZmNIT1* (NP_001105582); *OsNIT4* (NRL4_ORYSJ); *SbNIT4A* (Sb04g026950); *ZmNIT2* (NP_001105196); *LaNIT4A* (AAT36331); *LaNIT4B* (ABB51980); *NtNIT4A* (TOBTNIT4A); *NtNIT4B* (TOBTNIT4B); *AtNIT1* (AT3G44310); *AtNIT2* (AT3G44300); *AtNIT3* (AT3G44320); *BrNIT-T1* (ABM55733); *BrNIT-T2* (ABM55734); *RrNITA* (BAA01994)

To identify putative poplar nitrilase genes, a BLASTP search against the *P. trichocarpa* genome [22, 23] (http://www.phytozome.net/poplar) was carried out using the biochemically well-characterized nitrilases AtNIT1 and AtNIT4 from *A. thaliana* as template sequences. This approach revealed four sequences (Potri.004G199600, Potri.006G207700, Potri.016G074200, Potri.009G161000) with similarity to known nitrilases from other plants. While Potri.004G199600, Potri.006G207700, and Potri.016G074200 encode full-length nitrilase-like proteins containing the highly conserved catalytic triad that consists of a glutamic acid residue, a lysine residue, and a cysteine residue [4], the peptide encoded by Potri.009G161000 is shorter and lacks about two thirds of the nitrilase domain including the catalytic triad (Fig. 3). Thus, Potri.009G161000 is most likely a pseudogene and was not further investigated in this study. The open reading frames (ORFs) of Potri.004G199600, Potri.006G207700, and Potri.016G074200 were designated as *PtNIT1*, *PtNIT2*, and *PtNIT3*, respectively, and the encoded proteins showed 78.6% (PtNIT1), 58.3% (PtNIT2), and 55.7% (PtNIT3) amino acid sequence similarity to AtNIT4. PtNIT1 had a similar length as other plant nitrilases, while PtNIT2 and PtNIT3 showed an extended N-terminus containing a TCP transcription factor-like domain (Fig. 3). A dendrogram analysis revealed a close relationship of *PtNIT1*, *PtNIT2*, and *PtNIT3* to nitrilase genes of the NIT4 group (Fig. 4).

To verify the database sequences, we tried to amplify and sequence the complete ORFs of *PtNIT1*, *PtNIT2*, and *PtNIT3* from cDNA made from undamaged and herbivore-damaged *P. trichocarpa* leaves. While amplification of *PtNIT1* was successful, amplification of full-length as well as N-terminal truncated versions of *PtNIT2* and *PtNIT3* resulted only in trace amounts of amplicons, which could not be cloned into the sequencing vector.

### *PtNIT1* is expressed in poplar leaves and upregulated upon herbivory

**Fig. 5:**
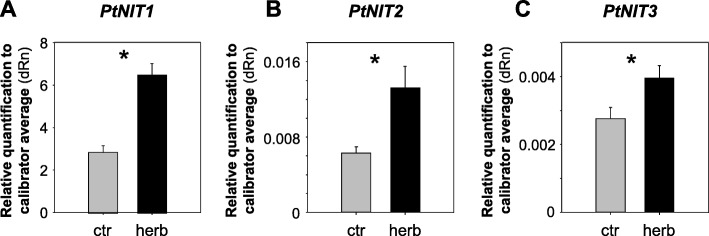
Transcript accumulation of *PtNIT1* (**a**), *PtNIT2* (**b**), and *PtNIT3* (**c**) in caterpillar-damaged (herb) and undamaged (ctr) leaves of *Populus trichocarpa*. Gene expression was analyzed using qRT-PCR and the relative quantification compared to a reference gene (ubiquitin) is shown. Asterisks indicate statistical significance in Student’s t-tests (*p* < 0.05). Means and SE are shown (*n* = 6 biological replicates)

The expression of *PtNIT1*, *PtNIT2*, and *PtNIT3* in undamaged and herbivore-damaged poplar leaves was analyzed using quantitative real time PCR (qRT-PCR). *PtNIT1* was found to be expressed in undamaged and herbivore-damaged leaves. However, herbivory by *L. dispar* caterpillars significantly increased its transcript abundance in comparison to the control treatment (Fig. 5). In contrast, *PtNIT2* and *PtNIT3* showed very low overall transcript abundance*,* although both genes were slightly upregulated upon herbivory.

### Recombinant PtNIT1 accepts β-cyanoalanine, benzyl cyanide, and diverse aromatic nitriles as substrate

To study the biochemical properties of PtNIT1, the complete ORF was inserted into the expression vector pET100/D-TOPO for heterologous expression in *Escherichia coli*. The recombinant His-tagged protein was Ni-purified from a bacterial protein extract and tested with different aromatic and aliphatic nitriles as substrates. Enzyme-catalyzed acid formation from different nitrile substrates was analyzed using LC-MS/MS. PtNIT1 showed enzymatic activity with all tested aromatic nitriles, including benzyl cyanide, 4-hydroxybenzyl cyanide, 3-phenylpropionitrile, and indole-3-acetonitrile, as well as with β-cyanoalanine (Table 1). A conversion of the aliphatic compounds butyronitrile, isovaleronitrile, and 3-butenenitrile, however, could not be detected. Notably, β-cyanoalanine was converted into aspartate and asparagine, suggesting that PtNIT1 possesses both nitrilase activity and nitrile-hydratase activity for this substrate. In contrast, formation of 2-phenylacetamide, the potential nitrile-hydratase activity product of benzyl cyanide, could not be observed.

**Table 1:** Activity of PtNIT1 with different aromatic and aliphatic nitriles as substrates. The purified recombinant protein was incubated with the potential nitrile substrate and the formation of the corresponding acids was analyzed using LC-MS/MS

substrate	substrate structure	activity
β-cyanoalanine		yes
3-phenylpropionitrile		yes
benzyl cyanide		yes
4-hydroxybenzyl cyanide		yes
indole-3-acetonitrile		yes
isovaleronitrile		no
3-butenenitrile		no
butyronitrile		no

A kinetic characterization of PtNIT1 revealed that the enzyme had a moderate affinity for β-cyanoalanine (*K*_m_ = 1.2 ± 0.1 mM), comparable to that of AtNIT4 [3]. The *K*_m_ values for benzyl cyanide (*K*_m_ = 24.7 ± 1.8 mM) and indole-3-acetonitrile (*K*_m_ = 10.0 ± 1.3 mM) were higher in comparison to the *K*_m_ value for β-cyanoalanine. However, the maximum velocity for the conversion of benzyl cyanide (*V*_max_ = 20.3 ± 0.7 nmol*mg^− 1^*min^− 1^) was about 10 times and 200 times higher in comparison to the velocity for β-cyanoalanine (*V*_max_ = 2.32 ± 0.08 nmol*mg^− 1^*min^− 1^) and indole-3-acetonitrile (*V*_max_ = 0.113 ± 0.007 nmol*mg^− 1^*min^− 1^), respectively. Hence, it is tempting to speculate that, while the conversion of β-cyanoalanine is the preferred reaction catalyzed by PtNIT1, this enzyme also plays a role in the metabolism of benzyl cyanide, which is highly abundant after herbivory in poplar leaves (Fig. 1).

### Putative ethylene biosynthesis genes are upregulated upon herbivory

**Fig. 6:**
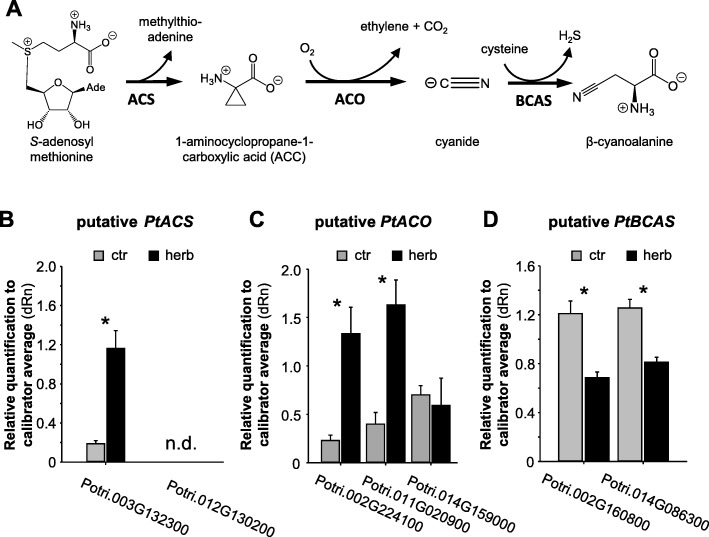
Transcript accumulation of genes potentially involved in ethylene and β-cyanoalanine biosynthesis in *Populus trichocarpa.* **a** Proposed biosynthetic pathway for β-cyanoalanine formation in poplar. ACS, 1-aminocyclopropane-1-carboxylic acid synthase; ACO, 1-aminocyclopropane-1-carboxylic acid oxidase; BCAS, β-cyanoalanine synthase. Gene expression of putative *PtACS* genes (**b**), *PtACO* genes (**c**), and *PtBCAS* genes (**d**) were measured using qRT-PCR in caterpillar-damaged (herb) and undamaged control (ctr) leaves of *Populus trichocarpa*. Transcript accumulation is shown as relative expression in comparison to ubiquitin. Asterisks indicate statistical significance in Student’s t-tests (*p* < 0.05). Means and SE are shown (*n* = 6 biological replicates). n.d., not detected

To test whether the accumulation of β-cyanoalanine in caterpillar-damaged poplar leaves (Fig. 1a) might be caused by an herbivore-induced upregulation of the ethylene biosynthesis pathway, we identified genes potentially involved in ethylene and β-cyanoalanine formation and tested their expression in undamaged and herbivore-damaged poplar leaves using qRT-PCR. Eleven putative ACC synthase genes (*PtACS*), seven putative ACC oxidase genes (*PtACO*), and two putative β-cyanoalanine synthase genes (*PtBCAS*) could be identified in the *P. trichocarpa* genome using a BLASTP analysis with characterized *ACS*, *ACO*, and *BCAS* genes from other plant species as templates (Additional file 1: Figures S1–S3). RNAseq data from undamaged and beetle-damaged *P. tremula* leaves provided by the *Populus* Genome Integrative Explorer PopGenIE v3 [24] (www.popgenie.org) revealed potentially herbivore-induced genes (Additional file 1: Figures S1–3). These genes, two putative *ACS* genes (Potri.003G132300, Potri.012G130200), three putative *ACO* genes (Potri.002G224100, Potri.014G159000, Potri.011G020900), and both putative *BCAS* genes (Potri.002G160800, Potri.014G086300), were further characterized by qRT-PCR in *L. dispar*-damaged and undamaged leaves of *P. trichocarpa*. One of the analyzed putative *ACS* genes and two out of the three *ACC* gene candidates showed significant upregulation after herbivory (Fig. 6). In contrast, the transcript accumulation of the two putative *BCAS* genes was significantly lower in herbivore-damaged leaves in comparison to undamaged control leaves (Fig. 6d).

### Analysis of potential PtNIT1 products in poplar leaves

**Fig. 7:**
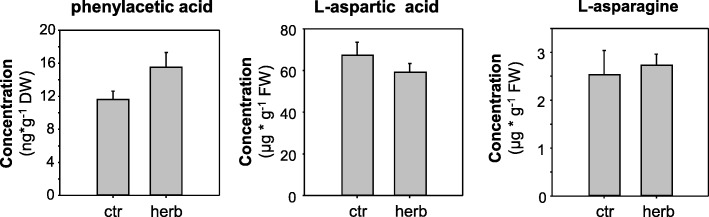
Accumulation of phenylacetic acid, L-aspartic acid, and L-asparagine in herbivore-damaged and undamaged *Populus trichocarpa* leaves. Methanol extracts made from *L. dispar-*damaged leaves (herb) and undamaged control leaves (ctr) were analyzed via LC-MS/MS. Means and SE are shown (*n* = 10 biological replicates). Phenylacetic acid, *p* = 0.066; Asp, *p* = 0.247; Asn, *p* = 0.121. DW, dry weight; FW, fresh weight

The potential products of the PtNIT1-catalyzed conversion of β-cyanoalanine and benzyl cyanide, aspartic acid and asparagine as well as phenylacetic acid, respectively, could also be detected in poplar leaves. However, in contrast to benzyl cyanide and β-cyanoalanine, their concentrations were not elevated after *L. dispar* caterpillar feeding (Fig. 7).

## Discussion

### The nitrilase gene family in *P. trichocarpa*

As many herbaceous plants, the tree species *P. trichocarpa* possesses a small nitrilase gene family consisting of three members (*PtNIT1*, *PtNIT2*, *PtNIT3*). Another poplar gene (Potri.009G161000) that appeared in our BLAST analysis also shows high similarity to known plant nitrilases, but encodes a protein lacking the catalytic triad as well as other conserved regions of plant nitrilases (Fig. 3). Thus, Potri.009G161000 was assumed to be a pseudogene. A dendrogram analysis revealed that *PtNIT1*, *PtNIT2*, and *PtNIT3* grouped together with *NIT4* homologues from other plants (Fig. 4). *PtNIT1* was expressed in herbivore-damaged leaves, suggesting a function in the catabolism of herbivore-induced nitriles such as β-cyanoalanine and benzyl cyanide. *PtNIT2* and *PtNIT3*, however, showed only trace expression and are thus most likely not involved in herbivore-induced nitrile metabolism in poplar leaves. In contrast to PtNIT1 and other plant nitrilases, PtNIT2 and PtNIT3 possess an extended N-terminus that was predicted as a TCP transcription factor domain (Fig. 3). Plant TCP transcription factors have been reported to be involved in the regulation of different growth and developmental processes such as the biogenesis of mitochondria [25], lateral branching [26], and early pollen development [27]. The TCP-like domains of PtNIT2 and PtNIT3 might have similar regulatory functions in poplar under certain conditions.

### PtNIT1 likely contributes to the catabolism of β-cyanoalanine in poplar

Biochemical characterization of recombinant PtNIT1 showed that the enzyme accepts β-cyanoalanine, benzyl cyanide, and indole-3-acetonitrile in vitro. However, considering the *K*_m_ and *V*_max_ values for the different substrates, PtNIT1 likely prefers β-cyanoalanine in vivo. This agrees with the substrate specificity of other nitrilases from the NIT4 group reported in the literature (Additional file 2: Table S1). Members of this group are in general designated β-cyanoalanine nitrile-hydratases/nitrilases, although they can also have activity towards aromatic nitriles such as indole-3-acetonitrile [8, 28] and benzyl cyanide [3].

The PtNIT1 substrate β-cyanoalanine was found to accumulate in herbivore-damaged poplar leaves (Fig. 1a). In plants, β-cyanoalanine is commonly produced as a detoxification product of hydrogen cyanide by β-cyanoalanine synthase (Fig. 6a). β-Cyanoalanine synthases catalyze the transfer of hydrogen cyanide to L-cysteine, resulting in the formation of β-cyanoalanine and hydrogen sulfide [29, 30]. Hydrogen cyanide itself can be formed as byproduct of the ethylene pathway [13] or as a breakdown product of cyanogenic compounds such as cyanogenic glycosides or cyanogenic lipids [14]. Since there is no evidence for the presence of cyanogenic glycosides or cyanogenic lipids in poplar [16], we propose that hydrogen cyanide is formed after herbivory in poplar as an ethylene byproduct. Indeed, many plant species produce and emit ethylene in response to herbivory [18]. Moreover, our qRT-PCR analysis showed that genes encoding enzymes involved in ethylene production, such as 1-aminocyclopropane-1-carboxylic acid synthase (ACS) and 1-aminocyclopropane-1-carboxylic acid oxidase (ACO), were significantly upregulated upon herbivory by gypsy moth caterpillars in poplar leaves (Fig. 6). Interestingly, the two putative β-cyanoalanine synthase genes Potri.002G16080 and Potri.014G086300 showed a small but significant down-regulation upon herbivory (Fig. 6d). Thus, hydrogen cyanide might accumulate to a certain level in herbivore-damaged poplar leaves before it is converted into β-cyanoalanine. Such ethylene-dependent accumulation of hydrogen cyanide could in general represent a defense reaction, repelling either the attacking caterpillar or inhibiting the growth of pathogens that use the wound site to enter the plant. It has been previously shown that hydrogen cyanide produced locally as an ethylene byproduct in pathogen-infested rice leaves contributes to resistance against the blast fungus *Magnaporthe oryzae* in rice [20].

### PtNIT1 might play a role in the formation of phenylacetic acid

Benzyl cyanide is one of the dominant herbivore-induced volatiles of poplar [16, 31, 32]. It is exclusively emitted from damaged leaves and showed a strong repellent effect against gypsy moth caterpillars in olfactometer experiments, suggesting a role in induced direct defence of poplar [17]. Here we showed that benzyl cyanide can also accumulate in significant amounts upon herbivory in poplar leaves (Fig. 1b). Although the *K*_m_ of recombinant PtNIT1 for benzyl cyanide was about twenty times higher in comparison to that for β-cyanoalanine, benzyl cyanide was found to accumulate in amounts thirty times higher than β-cyanoalanine in herbivore-damaged leaves (Fig. 1). This and a high maximum velocity for benzyl cyanide indicate that it can also act as substrate for PtNIT1 in planta. Indeed, feeding of poplar leaves with [α-^13^C]benzyl cyanide resulted in the formation of [α-^13^C]phenylacetic acid (Fig. 2), indicating a nitrilase activity in planta.

Phenylacetic acid belongs to the auxins [33–35] and has been detected in a variety of higher plants [36]. It regulates similar auxin-responsive genes as indole-3-acetic acid [15] but its activity is lower in comparison to that of indole-3-acetic acid [33]. Sugawara and colleagues [35] showed that the concentration of phenylacetic acid in Arabidopsis rosette leaves was much lower than in the roots, suggesting that phenylacetic acid functions mainly in the roots. Another Brassicales species, *Tropaeolum majus*, has been shown to accumulate phenylacetic acid exclusively in the roots where it can stimulate root growth [34]. In poplar, phenylacetic acid could be detected in undamaged and herbivore-damaged poplar leaves, but it was also found in leaf exudates (Fig. 2 f-g). Thus, it is tempting to speculate that this compound is transported through the phloem into the roots and might stimulate root rearrangement and/or growth upon aboveground herbivory. However, since we used detached leaves, the observed accumulation of phenylacetic acid in the water might also be caused by a passive exudation of this compound from the wounding site of the petiole. In contrast to benzyl cyanide, the accumulation of phenylacetic acid was not significantly influenced by the herbivore treatment (Fig. 2 and Fig. 7) and thus it is still unclear whether PtNIT1 contributes significantly to the formation of this hormone in planta. Although PtNIT1 was able to convert indole-3-acetonitrile into indole-3-acetic acid in vitro, the high *K*_m_ value and the very low *V*_max_ value in combination with only trace accumulation of indole-3-acetonitrile in poplar leaves [16] suggest at most only a minor role of this activity in the formation of indole-3-acetic acid in planta.

## Conclusion

Plant nitrilases are tightly associated with the metabolism of nitriles as has been shown for plants containing glucosinolates or cyanogenic glycosides. The present study investigated nitrilases in *P. trichocarpa*, a plant species that does not contain either of these potential nitrile sources, but accumulates the nitriles β-cyanoalanine and benzyl cyanide upon herbivory. PtNIT1 was identified as the major nitrilase expressed in poplar leaves and our data indicate that PtNIT1 is likely to be involved in the catabolism of herbivore-induced β-cyanoalanine and benzyl cyanide.

## Methods

### Plant and insect material and plant treatment

*Populus trichocarpa* plants were grown from monoclonal stem cuttings (genotype Muhle-Larsen, P&P Baumschule, Eitelborn, Germay) in a greenhouse (24 °C, 60% relative humidity, 16 h/8 h light/dark cycle) in a 1:1 mixture of sand and soil (Klasmann potting substrate; Klasmann-Deilmann, Geeste, Germany), until they reached 1 m in height, which is equal to 2.5 months of growth under greenhouse conditions.

Gypsy moth (*Lymantria dispar*) egg batches were provided by Hannah Nadel, United States Department of Agriculture Animal and Plant Health Inspection Service. After hatching, the caterpillars were reared on an artificial diet (gypsy moth diet; MP Biomedicals) until L4 stage. Caterpillars were starved in single cups for 1 day prior to the experiment.

For herbivore treatment of poplar, plants were enclosed in a PET bag (“Bratschlauch”, Toppits, Minden, Germany) with (herbivory treatment) or without (control treatment) 10 *L. dispar* caterpillars (larval stage, L4) for 20 h (start of the treatment, ~ 4 pm of day 1; end of the treatment, ~ 12 pm of day 2).

### [α-^13^C]benzyl cyanide treatment

Single leaves of *P. trichocarpa* (leaf plastochron index LPI 5) were cut and immediately placed in 5 ml sterile tap water. Each leaf was separately incubated in a closed desiccator for one day (starting at 8 am) in a 16 h light - 8 h dark cycle and treated with two *L. dispar* caterpillars (L3). Control leaves were incubated in the same setup without caterpillars. After removing the caterpillars, petioles were cut 1 mm above the original cutting site and the leaves were incubated for 24 h in the same desiccator with a volatile dispenser system consisting of a closed GC glass vial with a small glass capillary in the lid. For the benzyl cyanide treatment, the dispenser was filled with 50 μl n-hexane containing 1 μl [α-^13^C]benzyl cyanide (benzyl cyanide-(cyano-^13^C) 99 atom % ^13^C, Sigma-Aldrich). As a control, 50 μl pure n-hexane were used.

### Plant tissue sampling, RNA extraction, and reverse transcription

Poplar leaf material was harvested immediately after the herbivore treatment or benzyl cyanide feeding, flash-frozen with liquid nitrogen, and stored at − 80 °C until further processing. After the frozen leaf material was ground in liquid nitrogen to a fine powder with a Vibratory Micro Mill (Pulverisette-0, Fritsch, Idar-Oberstein, Germany), total RNA was isolated using an InviTrap Spin Plant RNA kit (Stratec, Berlin, Germany) according to the manufacturer’s instructions. RNA concentration, purity, and quality were assessed using a spectrophotometer (NanoDrop 2000c, Thermo Scientific, Wilmington, DE, USA) and an Agilent 2100 Bioanalyzer. Prior to cDNA synthesis, 1 μg of RNA was DNase treated using 1 U of DNase (ThermoFisher Scientific, https://www.thermofisher.com). Single-stranded cDNA was prepared using SuperScriptTM III reverse transcriptase and oligo (dT12–18) primers (ThermoFisher Scientific).

### Identification and isolation of nitrilase genes

Poplar nitrilase genes were identified using a BLASTP search against the *P. trichocarpa* genome database [22, 23] (http://www.phytozome.net/poplar) using Arabidopsis AtNIT1 (AEE77889.1) and AtNIT4 (AED93008.1) as template. The complete ORF of *PtNIT1* was amplified from cDNA made from *L. dispar*-damaged *P. trichocarpa* leaves and inserted into the pCR™-Blunt II-TOPO® vector (ThermoFisher Scientific). Primer sequence information is available in Additional file 2: Table S2.

### qRT-PCR analysis

cDNA was prepared as described above and diluted 1:10 with water. For the amplification of *nitrilase, ACC, ACO,* and *BCAS* gene fragments with a length of about 100–150 bp, primer pairs were designed having a Tm ≥ 60 °C, a GC content between 40 and 55%, and a primer length in the range of 20–25 nt (see Additional file 2: Table S2 for primer information). Primer specificity was confirmed by agarose gel electrophoresis, melting curve analysis, and standard curve analysis. Samples were run in triplicate using Brilliant III SYBR Green QPCR Master Mix (Agilent). The following PCR conditions were applied for all reactions: initial incubation at 95 °C for 3 min followed by 40 cycles of amplification (95 °C for 20 s and 60 °C for 20 s). SYBR Green fluorescence was measured at the end of each amplification cycle. Melting curves were recorded within a range of 55 to 95 °C at the end of a total of 40 cycles. Individual primer pair efficiency was tested by measuring Ct-values in stepwise diluted cDNA. Amplicon identity was validated via sequencing at the end of the run. All samples were run on the same PCR machine (MxPro – Mx3000P, Stratagene, Agilent Technologies, USA) in an optical 96-well plate for 6 biological replicates and 2 treatments. The relative normalized expression compared to the housekeeping gene *ubiquitin* [16] was analyzed using the Bio-Rad CFX Manager 3.1. The analysis of five potential housekeeping genes including *ubiquitin*, *actin* [37], *EF1α*, *histone,* and *tubulin* [38] showed that *ubiquitin* was the most stable one within the entity of all samples and was not influenced by the herbivore treatment (Additional file 2: Table S3).

### Dendrogram analysis and protein sequence alignment

A nucleotide sequence alignment of the poplar nitrilase genes and nitrilase genes from other plants was constructed with the Muscle (codon) algorithm (gap open, − 2.9; gap extend, 0; hydrophobicity multiplier, 1.5; clustering method, upgmb) implemented in MEGA6 [39]. The tree was reconstructed with MEGA6 using the Maximum Likelihood method (model/method, Kimura-2-parameter model; substitutions type, nucleotide; rates among sites, uniform rates; gaps/missing data treatment, partial deletion; site coverage cutoff, 80%) and a bootstrap resampling analysis with 1000 replicates was performed to evaluate the tree topology. An amino acid alignment of the poplar nitrilases was constructed and visualized with BioEdit (http://www.mbio.ncsu.edu/bioedit/bioedit.html) and the ClustalW algorithm.

### Heterologous expression of PtNIT1

For expression in *Escherichia coli*, the complete ORF of *PtNIT1* was subcloned into the pET100/D-TOPO® vector (N-terminal His-tag) and introduced in *E. coli* BL21(DE3) cells (Fisher Scientific). Cells were grown in TB-medium (1.2% *w*/*v* tryptone, 2.4% w/v yeast extract, 0.5% *v*/v glycerol, adjusted to pH 7.2 via potassium buffer, 50 μg/mL Carbenicillin) at 37 °C and 220 rpm until an OD600 of 0.4 was reached. The expression of *PtNIT1* was induced by adding 0.3 mM IPTG and the culture was grown over night at 32 °C and continuous shaking at 220 rpm. Cells were harvested by centrifugation (5000 x g, 10 min, 4 °C), the supernatant was decanted, and the pellet was resuspended in chilled lysis buffer (100 mM NaCl, 50 mM Tris-HCl, 2% v/v glycerol, 20 mM imidazole, pH 7.2, 10 mM MgCl_2,_ 0.3 mg/mL lysozyme, 100 u/L Benzonase (Fisher Scientific), 1 g/L proteinase inhibitor HP (Serva, www.serva.de)). The cells were disrupted by five freeze and thaw cycles and a 3 × 30 s treatment with a sonicator (Bandelin UW2070, Berlin, Germany; 70% intensity). The slurry was pelleted via centrifugation (12,000 x g, 20 min, 4 °C) and the supernatant was transferred to a new reaction tube. Soluble protein was purified from the supernatant using a Poly-Prep® Chromatography gravity flow column (Bio-Rad, www-bio-rad.com) with 500 μL HisPur™ Cobalt Resin (ThermoFischer Scientific) according to the manufacturer’s instructions (equilibration buffer/ washing buffer: 100 mM NaCl, 50 mM Tris-HCl, 2% v/v glycerol, 20 mM imidazole, pH 7.2; elution buffer: 100 mM NaCl, 50 mM Tris-HCl, 2% v/v glycerol; 150 mM imidazole, pH 7.2). The enzyme-containing eluate was desalted via an illustra NAP-10 gravity flow column (GE Healthcare) into a storage buffer (100 mM NaCl, 50 mM Tris-HCl, 5% v/v glycerol, pH 7.2).

Protein concentration was determined via a PCA-enhanced Biuret assay as stated by the manufacturer (Roti®-Quant universal, Karl Roth GmbH, Germany). Protein purity and identity was confirmed via SDS-PAGE and Western Blot with a His-Tag® antibody HRP conjugate (Novagen/ Merck Millipore).

### Nitrilase enzyme assays

Enzyme assays were performed in 1.5 ml glass vials containing 10 ng of purified enzyme and 1 mM of substrate in a total volume of 300 μL of potassium phosphate buffer (pH 7.2). The reaction was carried out for 1 h under shaking at 500 rpm and 32 °C and was stopped by adding 300 μl of 100% methanol. After placing on ice for 30 min, the denatured enzyme was removed by centrifugation (4.000 x g, 4 °C, 10 min) and the supernatant was transferred into a new glass vial or a 96 Deep-Well Agilent 41 mm plate for LC-MS/MS analysis of the reaction products.

To determine the kinetic parameters of PtNIT1, assays were carried out in triplicate with different substrate concentrations. The enzyme concentration and the incubation times were chosen so that the reaction velocity was linear during the incubation time period.

### LC-MS/MS analysis of poplar methanol extracts and nitrilase enzyme assays

Metabolites were extracted from ground plant material with methanol (10:1 *v*/*w*). Analytes were separated using an Agilent 1200 HPLC system on a Zorbax Eclipse XDB-C18 column (50 × 4.6 mm, 1.8 μm (Agilent Technologies)). The mobile phase consisted of formic acid (0.05% *v*/v) in water (A) and acetonitrile (B). The column temperature was maintained at 25 °C. LC-MS parameters are given in Additional file 2: Table S4.

The liquid chromatography was coupled to an API5000 tandem mass spectrometer (Applied Biosystems) equipped with a Turbospray ion source (ion spray voltage, 5500 eV; turbo gas temperature, 700 °C; nebulizing gas, 70 p.s.i.; curtain gas, 35 p.s.i.; heating gas, 70 p.s.i.). Multiple reaction monitoring (MRM) was used to monitor a parent ion → product ion reaction for each analyte. Detailed parameters are given in Additional file 2: Table S5. If not stated otherwise, analytes were quantified via external standard curves of consecutive 50% dilutions, starting from a 1 μg/ml concentration to a concentration of 1.95 ng/ml. External standards were diluted in aqueous methanol (50% v/v) (compound information and suppliers are listed in Additional file 2: Table S6).

Amino acids produced in enzyme assays by recombinant PtNIT1 were derivatized with 9-fluorenylmethoxy-carbonyl chloride (FMOC-Cl) (Fluka, Germany). A 100 μl portion of the extract was mixed with 100 μl of borate buffer (0.8 M, pH 8.0) spiked with internal standard amino acid mix (^13^C-, ^15^N-labelled amino acids (algal amino acids ^13^C, ^15^N, Isotec, Miamisburg, USA) at a concentration of 20 μg of the algal amino acid mix per mL of borate buffer). A 200 μl portion of FMOC-Cl reagent (30 mM in acetonitrile) was added to the samples and mixed. After 5 min, 800 μl of hexane was added to stop the reaction and to remove excess FMOC-Cl reagent, mixed and let stand for the separation of liquid phases. A 200 μl portion of the aqueous liquid phase was then transferred to a 96 deep well plate for LC-MS analysis (chromatographic gradient C, Additional file 2: Table S4). MRM details are given in Additional file 2: Table S5. Analyst 1.5 software (Applied Biosystems, Darmstadt, Germany) was used for data acquisition and processing.

### GC-MS/FID analysis of poplar hexane extracts

For the analysis of benzyl cyanide accumulation in poplar leaves, 100 mg of leaf powder was extracted in a GC glass vial with 400 μl hexane including 10 ng/uL nonyl acetate as internal standard. The extracts were shaken for one hour at 300 rpm. After centrifugation for 10 min at 5,000 x g, the supernatant was analyzed using gas chromatography-mass spectrometry (GC-MS).

A Hewlett-Packard model 6890 gas chromatograph was employed with He (MS) or H_2_ (FID) as carrier gas at 2 ml min^− 1^, splitless injection (injector temperature, 230 °C; injection volume, 1 μL) and a DB-5MS column (Agilent, Santa Clara, USA, 30 m × 0.25 mm × 0.25 μm). The GC oven temperature was held for 2 min at 45 °C and then increased to 225 °C with a gradient of 6 °C min^− 1^, held for another 2 min, and then further increased to 250 °C with 100 °C min^− 1^ and a hold for 1 min. The coupled mass spectrometer was a Hewlett-Packard model 5973 with a quadrupole mass selective detector (transfer line temperature, 230 °C; source temperature, 230 °C; quadrupole temperature, 150 °C; ionization potential, 70 eV; scan range of 50–400 amu). Benzyl cyanide was identified using an authentic standard (Sigma Aldrich, www.sigmaaldrich.com). Quantification was performed with the trace of a flame ionization detector (FID) operated at 300 °C. The peak area of benzyl cyanide was compared with that of the internal standard assuming equal response factors. The concentration of [α-^13^C]benzyl cyanide was quantified via GC-MS and an external standard curve. For quantification, the molecular ion *m/z* 118 was used.

### Accession numbers

Sequence data for poplar nitrilase 1 (*PtNIT1*) can be found in GenBank under the identifier MF182356.

## Additional files

Additional file 1: **Figure S1.** The ACC synthase (ACS) gene family in *Populus trichocarpa*. **Figure S2**. The ACC oxidase (ACO) gene family in *Populus trichocarpa*. **Figure S3.** The β-cyanoalanine synthase (BCAS) gene family in *Populus trichocarpa*. (PPTX 127 kb)

Additional file 2: **Table S1.** Comparison of nitrile hydratase and nitrilase activity of characterized plant nitrilases. **Table S2.** Oligonucleotides used for isolation and qRT-PCR analysis of poplar nitrilase genes. **Table S3.** Expression levels of potential housekeeping genes in herbivore-induced (herb) and untreated control (ctr) leaves of *P. trichocarpa*. **Table S4.** HPLC conditions (gradients) used for separation and analysis of nitrilase substrates/products. **Table S5.** MS/MS parameters used for LC-MS/MS analysis on API5000 triple quad mass spectrometer. **Table S6.** Compounds used as substrates for enzyme assays and standards for LC-MS/MS and GC-MS quantification. (DOCX 44 kb)

## Abbreviations

BCA: β-cyanoalanine
DW: Dry weight
FW: Fresh weight
IAA: Indole-3-acetic acid
IAN: Indole-3-acetonitrile
PAA: Phenylacetic acid
