# Supplementary material for: The nitrilase PtNIT1 catabolizes herbivore-induced nitriles in Populus trichocarpa
Source: BMC Plant Biol. 2018 Oct 22;18:251. doi: 10.1186/s12870-018-1478-z (PMC6196558; doi:10.1186/s12870-018-1478-z)
Supplement: Supplementary file 1 — Figure S1. The ACC synthase (ACS) gene family in Populus trichocarpa. Figure S2. The ACC oxidase (ACO) gene family in Populus trichocarpa. Figure S3. The β-cyanoalanine synthase (BCAS) gene family in Populus trichocarpa. (PPTX 127 kb) [file 12870_2018_1478_MOESM1_ESM.pptx]

## Slide 1
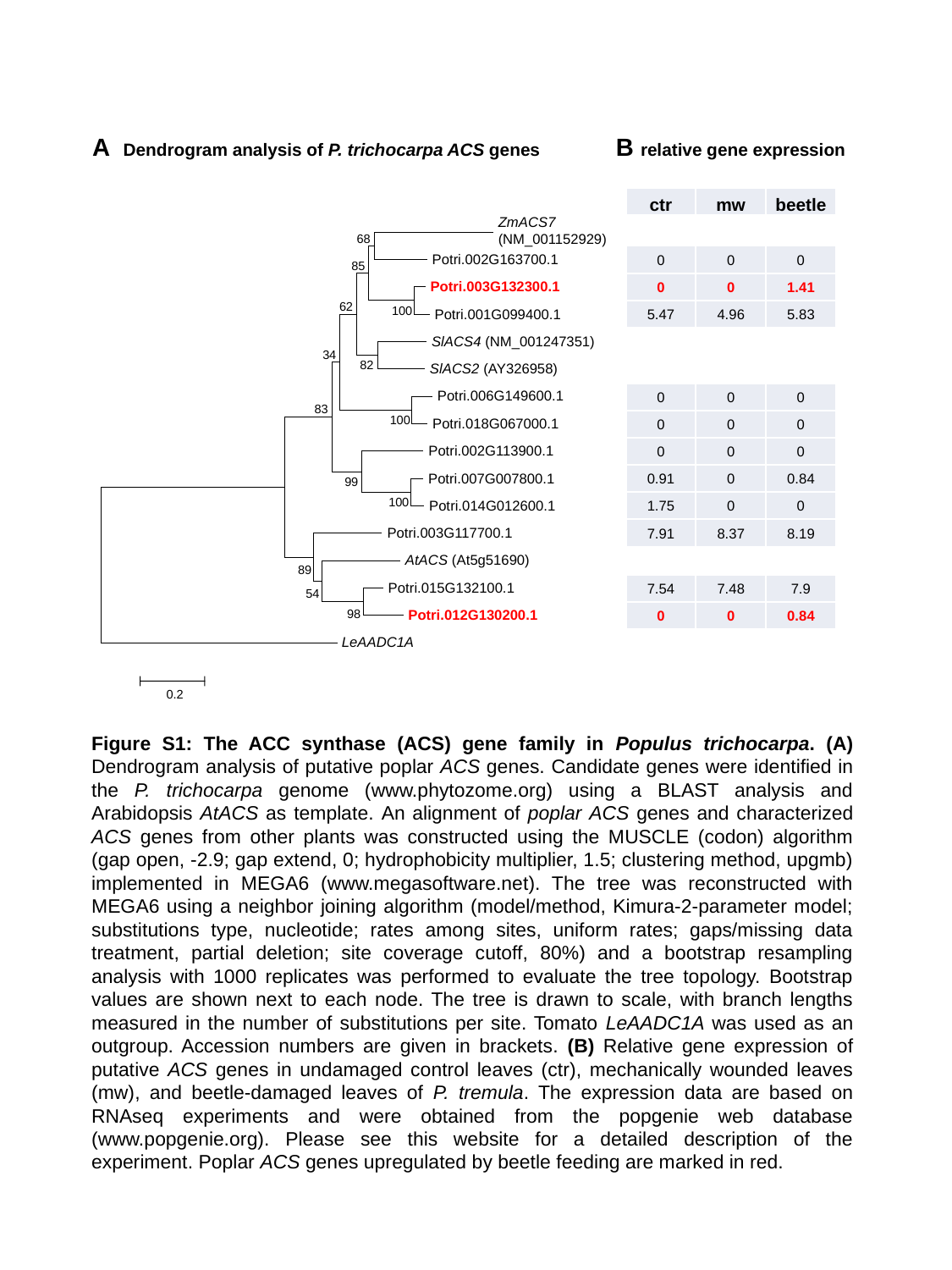

A Dendrogram analysis of P. trichocarpa ACS genes
B relative gene expression
| ctr | mw | beetle |
| --- | --- | --- |
 ZmACS7
 (NM_001152929)
68
| 0 | 0 | 0 |
| --- | --- | --- |
| 0 | 0 | 1.41 |
| 5.47 | 4.96 | 5.83 |
 Potri.002G163700.1
85
 Potri.003G132300.1
62
100
 Potri.001G099400.1
 SlACS4 (NM_001247351)
34
82
 SlACS2 (AY326958)
| 0 | 0 | 0 |
| --- | --- | --- |
| 0 | 0 | 0 |
| 0 | 0 | 0 |
| 0.91 | 0 | 0.84 |
| 1.75 | 0 | 0 |
| 7.91 | 8.37 | 8.19 |
 Potri.006G149600.1
83
100
 Potri.018G067000.1
 Potri.002G113900.1
 Potri.007G007800.1
99
100
 Potri.014G012600.1
 Potri.003G117700.1
 AtACS (At5g51690)
89
| 7.54 | 7.48 | 7.9 |
| --- | --- | --- |
| 0 | 0 | 0.84 |
 Potri.015G132100.1
54
 Potri.012G130200.1
98
 LeAADC1A
0.2
Figure S1: The ACC synthase (ACS) gene family in Populus trichocarpa. (A) Dendrogram analysis of putative poplar ACS genes. Candidate genes were identified in the P. trichocarpa genome (www.phytozome.org) using a BLAST analysis and Arabidopsis AtACS as template. An alignment of poplar ACS genes and characterized ACS genes from other plants was constructed using the MUSCLE (codon) algorithm (gap open, -2.9; gap extend, 0; hydrophobicity multiplier, 1.5; clustering method, upgmb) implemented in MEGA6 (www.megasoftware.net). The tree was reconstructed with MEGA6 using a neighbor joining algorithm (model/method, Kimura-2-parameter model; substitutions type, nucleotide; rates among sites, uniform rates; gaps/missing data treatment, partial deletion; site coverage cutoff, 80%) and a bootstrap resampling analysis with 1000 replicates was performed to evaluate the tree topology. Bootstrap values are shown next to each node. The tree is drawn to scale, with branch lengths measured in the number of substitutions per site. Tomato LeAADC1A was used as an outgroup. Accession numbers are given in brackets. (B) Relative gene expression of putative ACS genes in undamaged control leaves (ctr), mechanically wounded leaves (mw), and beetle-damaged leaves of P. tremula. The expression data are based on RNAseq experiments and were obtained from the popgenie web database (www.popgenie.org). Please see this website for a detailed description of the experiment. Poplar ACS genes upregulated by beetle feeding are marked in red.

## Slide 2
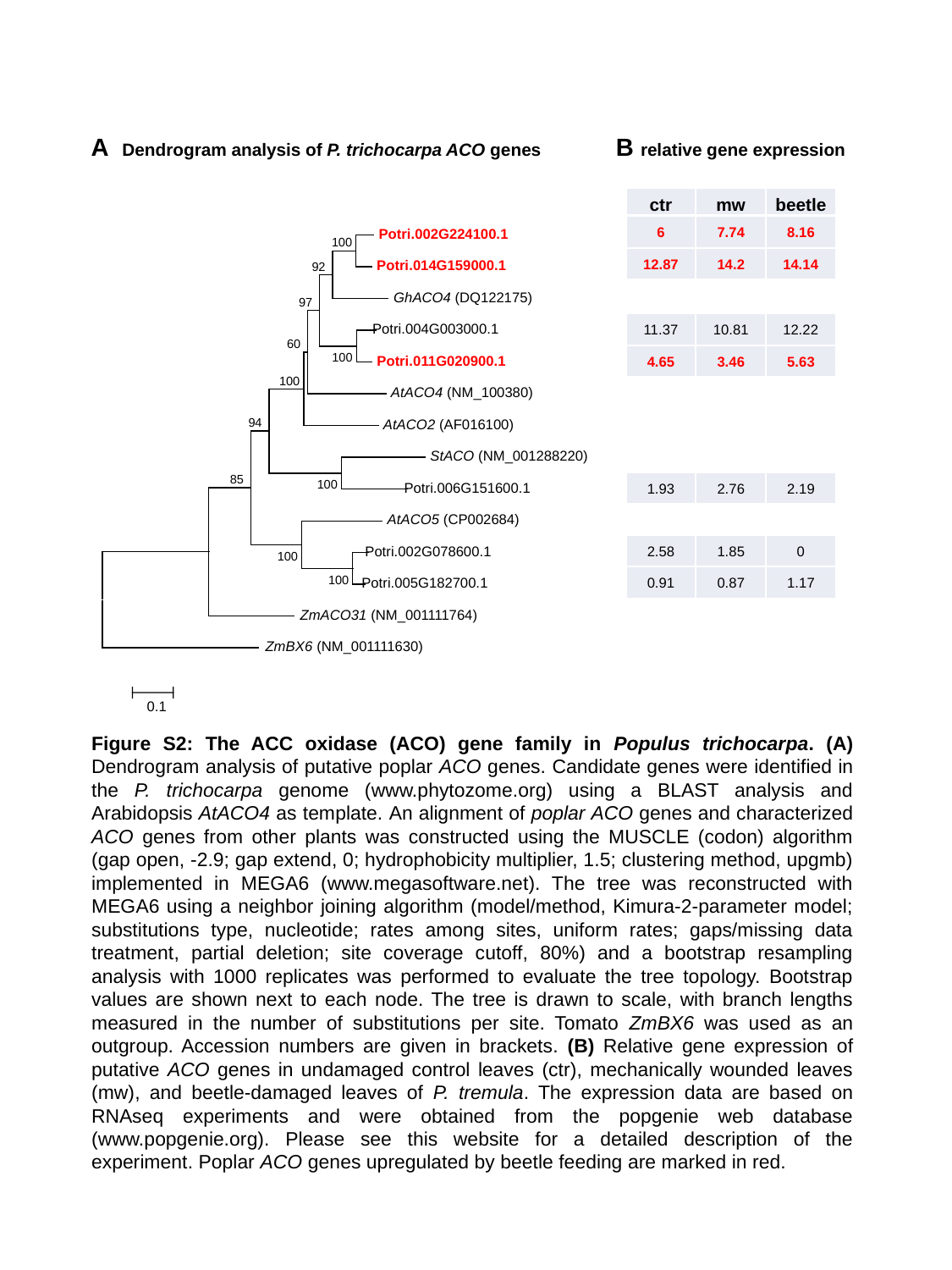

A Dendrogram analysis of P. trichocarpa ACO genes
B relative gene expression
| ctr | mw | beetle |
| --- | --- | --- |
| 6 | 7.74 | 8.16 |
| --- | --- | --- |
| 12.87 | 14.2 | 14.14 |
 Potri.002G224100.1
100
 Potri.014G159000.1
92
 GhACO4 (DQ122175)
97
| 11.37 | 10.81 | 12.22 |
| --- | --- | --- |
| 4.65 | 3.46 | 5.63 |
 Potri.004G003000.1
60
100
 Potri.011G020900.1
100
 AtACO4 (NM_100380)
94
 AtACO2 (AF016100)
 StACO (NM_001288220)
85
| 1.93 | 2.76 | 2.19 |
| --- | --- | --- |
100
 Potri.006G151600.1
 AtACO5 (CP002684)
| 2.58 | 1.85 | 0 |
| --- | --- | --- |
| 0.91 | 0.87 | 1.17 |
 Potri.002G078600.1
100
100
 Potri.005G182700.1
 ZmACO31 (NM_001111764)
 ZmBX6 (NM_001111630)
0.1
Figure S2: The ACC oxidase (ACO) gene family in Populus trichocarpa. (A) Dendrogram analysis of putative poplar ACO genes. Candidate genes were identified in the P. trichocarpa genome (www.phytozome.org) using a BLAST analysis and Arabidopsis AtACO4 as template. An alignment of poplar ACO genes and characterized ACO genes from other plants was constructed using the MUSCLE (codon) algorithm (gap open, -2.9; gap extend, 0; hydrophobicity multiplier, 1.5; clustering method, upgmb) implemented in MEGA6 (www.megasoftware.net). The tree was reconstructed with MEGA6 using a neighbor joining algorithm (model/method, Kimura-2-parameter model; substitutions type, nucleotide; rates among sites, uniform rates; gaps/missing data treatment, partial deletion; site coverage cutoff, 80%) and a bootstrap resampling analysis with 1000 replicates was performed to evaluate the tree topology. Bootstrap values are shown next to each node. The tree is drawn to scale, with branch lengths measured in the number of substitutions per site. Tomato ZmBX6 was used as an outgroup. Accession numbers are given in brackets. (B) Relative gene expression of putative ACO genes in undamaged control leaves (ctr), mechanically wounded leaves (mw), and beetle-damaged leaves of P. tremula. The expression data are based on RNAseq experiments and were obtained from the popgenie web database (www.popgenie.org). Please see this website for a detailed description of the experiment. Poplar ACO genes upregulated by beetle feeding are marked in red.

## Slide 3
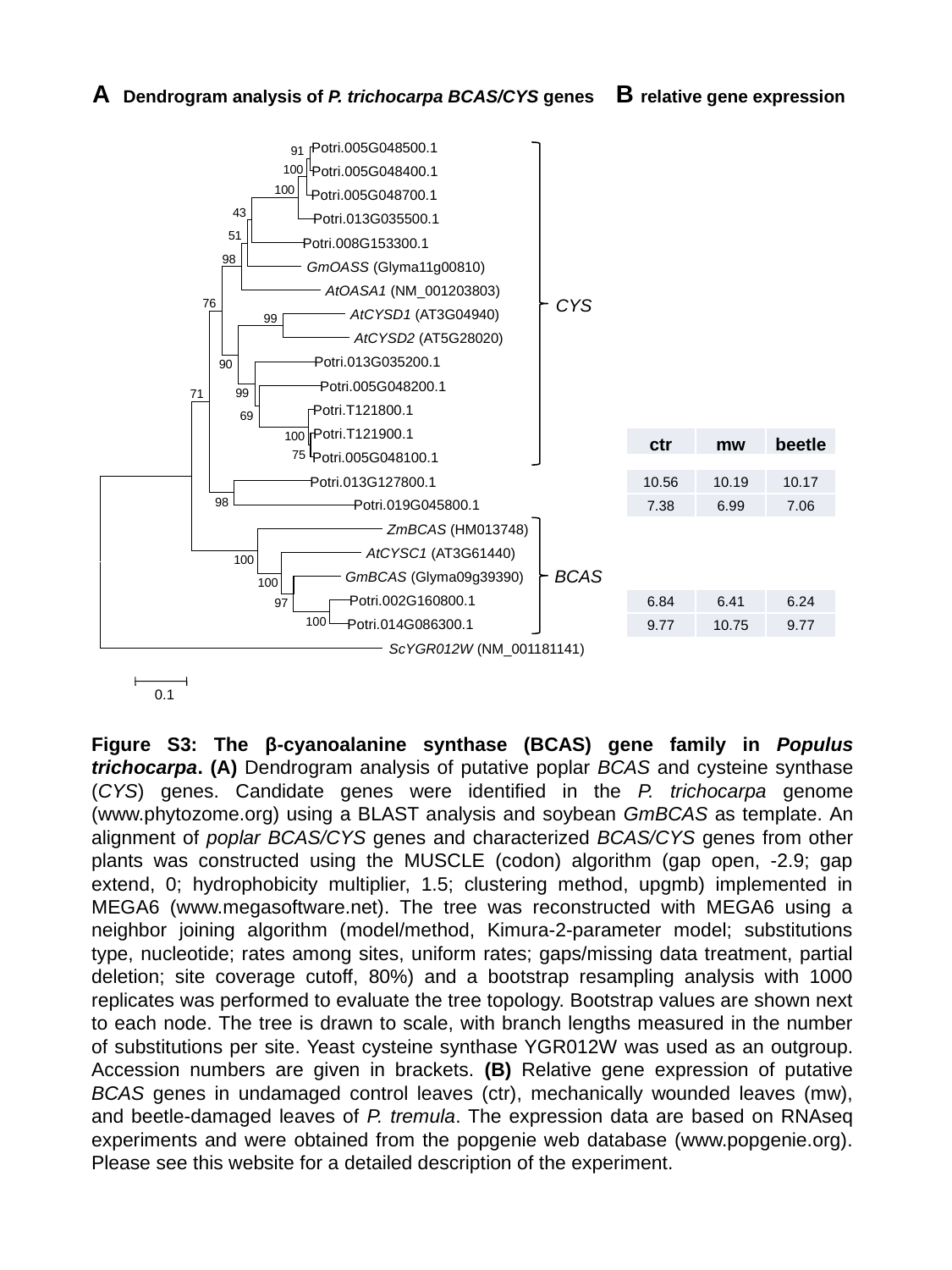

A Dendrogram analysis of P. trichocarpa BCAS/CYS genes
B relative gene expression
 Potri.005G048500.1
91
100
 Potri.005G048400.1
100
 Potri.005G048700.1
43
 Potri.013G035500.1
51
 Potri.008G153300.1
98
 GmOASS (Glyma11g00810)
 AtOASA1 (NM_001203803)
CYS
76
 AtCYSD1 (AT3G04940)
99
 AtCYSD2 (AT5G28020)
 Potri.013G035200.1
90
 Potri.005G048200.1
99
71
 Potri.T121800.1
69
 Potri.T121900.1
| ctr | mw | beetle |
| --- | --- | --- |
100
75
 Potri.005G048100.1
| 10.56 | 10.19 | 10.17 |
| --- | --- | --- |
| 7.38 | 6.99 | 7.06 |
 Potri.013G127800.1
98
 Potri.019G045800.1
 ZmBCAS (HM013748)
 AtCYSC1 (AT3G61440)
100
BCAS
 GmBCAS (Glyma09g39390)
100
| 6.84 | 6.41 | 6.24 |
| --- | --- | --- |
| 9.77 | 10.75 | 9.77 |
 Potri.002G160800.1
97
100
 Potri.014G086300.1
 ScYGR012W (NM_001181141)
0.1
Figure S3: The β-cyanoalanine synthase (BCAS) gene family in Populus trichocarpa. (A) Dendrogram analysis of putative poplar BCAS and cysteine synthase (CYS) genes. Candidate genes were identified in the P. trichocarpa genome (www.phytozome.org) using a BLAST analysis and soybean GmBCAS as template. An alignment of poplar BCAS/CYS genes and characterized BCAS/CYS genes from other plants was constructed using the MUSCLE (codon) algorithm (gap open, -2.9; gap extend, 0; hydrophobicity multiplier, 1.5; clustering method, upgmb) implemented in MEGA6 (www.megasoftware.net). The tree was reconstructed with MEGA6 using a neighbor joining algorithm (model/method, Kimura-2-parameter model; substitutions type, nucleotide; rates among sites, uniform rates; gaps/missing data treatment, partial deletion; site coverage cutoff, 80%) and a bootstrap resampling analysis with 1000 replicates was performed to evaluate the tree topology. Bootstrap values are shown next to each node. The tree is drawn to scale, with branch lengths measured in the number of substitutions per site. Yeast cysteine synthase YGR012W was used as an outgroup. Accession numbers are given in brackets. (B) Relative gene expression of putative BCAS genes in undamaged control leaves (ctr), mechanically wounded leaves (mw), and beetle-damaged leaves of P. tremula. The expression data are based on RNAseq experiments and were obtained from the popgenie web database (www.popgenie.org). Please see this website for a detailed description of the experiment.
