# Supplementary material for: The nitrilase PtNIT1 catabolizes herbivore-induced nitriles in Populus trichocarpa
Source: BMC Plant Biol. 2018 Oct 22;18:251. doi: 10.1186/s12870-018-1478-z (PMC6196558; doi:10.1186/s12870-018-1478-z)
Supplement: Supplementary file 2 — Table S1. Comparison of nitrile hydratase and nitrilase activity of characterized plant nitrilases. Table S2. Oligonucleotides used for isolation and qRT-PCR analysis of poplar nitrilase genes. Table S3. Expression levels of potential housekeeping genes in herbivore-induced (herb) and untreated control (ctr) leaves of P. trichocarpa. Table S4. HPLC conditions (gradients) used for separation and analysis of nitrilase substrates/products. Table S5. MS/MS parameters used for LC-MS/MS analysis on API5000 triple quad mass spectrometer. Table S6. Compounds used as substrates for enzyme assays and standards for LC-MS/MS and GC-MS quantification. (DOCX 44 kb) [file 12870_2018_1478_MOESM2_ESM.docx]

**Table S1**

**Comparison of nitrile hydratase and nitrilase activities of characterized plant nitrilases.** The Vmax ratios ± SE are given.

| **Enzyme** | ***V*_max_ nitrile hydratase/*V*_max_ nitrilase** |
| --- | --- |
| LaNIT4A | 4.02 ± 0.17 ^a^ |
| LaNIT4B | 3.34 ± 0.12 ^a^ |
| AtNIT4 | 1.36 ± 0.21 ^b^ |
| NtNIT4A | 0.87 ± 0.04 ^b^ |
| NtNIT4B | 1.06 ± 0.12 ^b^ |
| PtNIT1 | 4.02 ± 0.002 ^c^ |

^a^ Piotrowski et al., 2006; ^b^ Piotrowski et al., 2001; ^c^ this study

**Table S2**

**Oligonucleotides used for isolation and qRT-PCR analysis of poplar nitrilase genes.**

| **Name** | **Sequence** | | **Usage** |
| --- | --- | --- | --- |
| PtNIT1-fwd  PtNIT1-rev  PtNIT2-fwd  PtNIT2-rev  PtNIT3-fwd  PtNIT3-rev  PtNIT1-fwd pET200  PtNIT1-rev pET200  PtNIT1fwd qPCR  PtNIT1rev qPCR  PtNIT2fwd qPCR  PtNIT2rev qPCR  PtNIT3fwd qPCR  PtNIT3rev qPCR  PtACO2-fwd  PtACO2-rev  PtACO14-fwd  PtACO14-rev  PtACO11-fwd  PtACO11-rev  PtACS1-fwd  PtACS1-rev  PtACS2-fwd  PtACS2-rev  PtACS3-fwd  PtACS3-rev  PtBCAS2-fwd  PtBCAS2-rev  PtBCAS14-fwd  PtBCAS14-rev  PtUBI-fwd  PtUBI-rev  PtACT-fwd  PtACT-rev  PtEF1a-fwd  PtEF1a-rev  PtHIS-fwd  PtHIS-rev  PtTUB-fwd  PtTUB-rev | | ATGCAAGCAACTGTACCAGTTTCATCATC  CTATTTACGAGAGGCTTCAGTTTTTGCTG  ATGGAGGAATCAACAAAAAATGCGG  TCACCTGACATGGTTCTAATTCCGCC ATGGAGGAAGAATTCGCAAA  TCACCCTGAAAGCTCTTCAAGA  CACCATGCAAGCAACTGTACCAG  CTCTTTACGAGAGGCTTCAGTTTTTGCTG  CTGCCACTCTAGATAAGGCTGAGA  CAACTTCTGGACCAGGAACATCAA  CGGCAACATTGGATAAAGCAGAGA  GTCTCTCAACTTCAGGACCAGGCA  TTGATGTGCCTGGTCCTGAAGTTG  TGTGGGTCATTGATGCTCTCCA  TTGGACACTGTGGAGAGAATGG  CAAGTGGAAGGTGCTCTCCC  AGAGCACCTTCCAGTTACGC  TTGGTGCCAAAGGTTGGACT  GGGTCCAAGGGACCGAATTT  CCGGTGCTGGATACATGACA  CTGATCCCCATCATCCCACG  CAGGACTGCAAATGGAGGCT  CGACCATCTTCAGCTCCCAG  TGAGGAGACCAGCCCAAAAC  TGAATGGAGGAGCTACCGGA  TGCCCAACGGATTTGATGGA  ACTCCTCGATCTTCACACGC  AGCAGAGGCAACAGTAGCAG  TTGGAATCCACTCCAGGTGC  GAGATACTTCCCGACGCCAG  GTTGATTTTTGCTGGGAAGC  GATCTTGGCCTTCACGTTGT  CCCATTGAGCACGGTATTGT  TACGACCACTGGCATACAGG  GGCAAGGAGAAGGTACACAT  CAATCACACGCTTGTCAATA  ACTGCTCGTAAGTCTACTGGAGG  GCGGTAACGGTGAGGCTTCTTC  GGAGGTGGAACTGGATCAGGAATG  GGCATTGTAAGGCTCAACCACTGT | ORF cloning  ORF cloning  ORF cloning  ORF cloning  ORF cloning  ORF cloning  expression  expression  qRT-PCR  qRT-PCR  qRT-PCR  qRT-PCR  qRT-PCR  qRT-PCR  qRT-PCR  qRT-PCR  qRT-PCR  qRT-PCR  qRT-PCR  qRT-PCR  qRT-PCR  qRT-PCR  qRT-PCR  qRT-PCR  qRT-PCR  qRT-PCR  qRT-PCR  qRT-PCR  qRT-PCR  qRT-PCR  qRT-PCR  qRT-PCR  qRT-PCR  qRT-PCR  qRT-PCR  qRT-PCR  qRT-PCR  qRT-PCR  qRT-PCR  qRT-PCR |

**Table S3**

|  | ***Ubiquitin*** | ***Actin*** | ***EF1-α*** | ***Histone*** | ***Tubuline*** |
| --- | --- | --- | --- | --- | --- |
| **ctr-1** | 18.27 | 19.84 | 19.45 | 18.21 | 21.53 |
| **ctr-2** | 17.35 | 20.58 | 20.26 | 17.91 | 22.47 |
| **ctr-3** | 17.90 | 20.74 | 20.45 | 18.79 | 21.64 |
| **ctr-4** | 18.80 | 21.36 | 21.13 | 19.40 | 23.18 |
| **ctr-5** | 19.21 | 21.12 | 20.62 | 19.37 | 23.17 |
| **ctr-6** | 18.60 | 21.36 | 21.18 | 19.15 | 22.95 |
| **herb-1** | 18.27 | 20.55 | 21.02 | 19.10 | 22.88 |
| **herb-2** | 19.72 | 22.13 | 22.47 | 20.44 | 24.17 |
| **herb-3** | 17.58 | 19.76 | 19.96 | 18.23 | 22.19 |
| **herb-4** | 17.66 | 20.24 | 20.62 | 18.38 | 22.36 |
| **herb-5** | 18.40 | 21.75 | 21.94 | 19.38 | 24.05 |
| **herb-6** | 18.49 | 20.85 | 21.18 | 19.24 | 22.82 |
| **mean ΔCq**  **STDEV** | 18.35  0.687 | 20.86  0.726 | 20.86  0.826 | 18.97  0.702 | 22.78  0.819 |

**Expression levels of potential house-keeping genes in herbivore-induced (herb) and untreated control (ctr) leaves of *Populus trichocarpa*.** The ΔCq values, their means, and the standard deviations (STDEV) are shown.

**Table S4**

**HPLC conditions (gradients) used for separation and analysis of nitrilase substrates/products.** See also Supplemental table S5 for MS/MS parameters.

|  | **Time** (min) | **Flow** (µl/min) | **Water + 0,05% formic acid** (%) | **Acetonitrile** (%) |
| --- | --- | --- | --- | --- |
| **Gradient A** | 0  1  2.7  3  3.1  6 | 1100  1100  1100  1100  1100  1100 | 97  97  0  0  97  97 | 3  3  100  100  3  3 |
| **Gradient B** | 0  0.5  4  4.02  5  5.02  7 | 1100  1100  1100  1100  1100  1100  1100 | 95  95  55  0  0  95  95 | 5  5  45  100  100  5  5 |
| **Gradient C** | 0  0.5  4.5  6  6.5  6.51  9 | 1100  1100  1100  1100  1100  1100  1100 | 90  90  10  0  0  90  90 | 10  10  90  100  100  10  10 |

**Table S5**

**MS/MS parameter used for LC-MS/MS analysis.** The details of the HPLC gradients indicated in the right column are given in Supplemental table S4.

| **compound** | **Q1** | **Q3** | **Scan time  (ms)** | **DP** | **CE** | **CXP** | **mode** | **HPLC  gradient** | **Retention time (min)** |
| --- | --- | --- | --- | --- | --- | --- | --- | --- | --- |
| (*E*/*Z*)-phenylacetaldoxime  2-phenylacetamide  phenylacetic acid  β-cyano-L-alanine  isovaleric acidbutyric acid  indole-3-acetic acid  phenylpropionic acid  4-hydroxy phenylacetic acid  3-butenoic acid  L-asparagine-FMOC  L-aspartic acid-FMOC  L-asparagine-FMOC-  (U-^13^C, ^15^N) | 136.1  136.1  134.854  113  101.1  87.1  173.85  149  151  85  353  354  359 | 119.1  119.1  91  96  101.1  87.1  129.9  105  107  85  157  157.8  163 | 15  15  1000  1000  15  15  15  15  15  15  400  400  400 | 56  56  -25  -50  -50  -50  -25  -50  -50  -50  -65  -70  -65 | 17  17  -10  -13  -5  -5  -14  -14  -15  -5  -12  -16  -12 | 4  4  -10  -5  -11  -11  -2  -5  -5  -11  -4  -4  -4 | pos  pos  neg  neg  neg  neg  neg  neg  neg  neg  neg  neg  neg | A  A  B  B  B  B  B  B B  B  C  C  C | 3.06/3.11  2.64  4.15  0.44  3.57  2.32  4.21  4.8  2.56  1.56  3.7  3.9  3.7 |

**Table S6**

**Compounds used as substrates for enzyme assays and standards
for LC-MS/MS and GC-MS quantification.**

| **compound** | **CAS** | **Supplier** |
| --- | --- | --- |
| 2-Phenylacetamide  Vinylacetic acid  Benzyl cyanide  4-Hydroxybenzyl cyanide  Benzyl cyanide-(cyano-13C)  Isovaleric acid  β-cyano-L-alanine  3-indoleacetonitrile  Indole-3-acetic acid  phenylpropionic acid  4-hydroxy phenylacetic acid  L-asparagine  L-aspartic acid  phenylacetic acid  butyric acid  3-butenoic acid  3-phenylpropionitrile  isovaleronitrile  3-butenenitrile  butyronitrile | 103-81-1  625-38-7  140-29-4  14191-95-8  83552-81-2  503-74-2  6232-19-5  771-51-7  87-51-4  501-52-0  156-38-7  5794-13-8  56-84-8  103-82-2  107-92-6  625-38-7  645-59  625-28-5  109-75-1  109-74-0 | Aldrich  Merck  Aldrich  Merck  Aldrich  Fluka  Santa Cruz  Riedel Merck  Duchefa  Sigma  Acros  Duchefa  Merck  Aldrich  Aldrich  Sigma  Merck  Aldrich  Fluka  Fluka |
